# A Cationic Surfactant-Decorated Liquid Crystal-Based Aptasensor for Label-Free Detection of Malathion Pesticides in Environmental Samples

**DOI:** 10.3390/bios11030092

**Published:** 2021-03-23

**Authors:** Duy Khiem Nguyen, Chang-Hyun Jang

**Affiliations:** Department of Chemistry, Gachon University, Seongnam-daero 1342, Sujeong-gu, Seongnam-si 13120, Gyeonggi-do, Korea; khiem80@gachon.ac.kr

**Keywords:** malathion, liquid crystals, liquid crystal biosensor, pesticides, DNA aptamer, polarized light microscopy

## Abstract

We report a liquid crystal (LC)-based aptasensor for the detection of malathion using a cationic surfactant-decorated LC interface. In this method, LCs displayed dark optical images when in contact with aqueous cetyltrimethylammonium bromide (CTAB) solution due to the formation of a self-assembled CTAB monolayer at the aqueous/LC interface, which induced the homeotropic orientation of LCs. With the addition of malathion aptamer, the homeotropic orientation of LCs changed to a planar one due to the interactions between CTAB and the aptamer, resulting in a bright optical image. In the presence of malathion, the formation of aptamer-malathion complexes caused a conformational change of the aptamers, thereby weakening the interactions between CTAB and the aptamers. Therefore, CTAB is free to induce a homeotropic ordering of the LCs, which corresponds to a dark optical image. The developed sensor exhibited high specificity for malathion determination and a low detection limit of 0.465 nM was achieved. Moreover, the proposed biosensor was successfully applied to detect malathion in tap water, river water, and apple samples. The proposed LC-based aptasensor is a simple, rapid, and convenient platform for label-free monitoring of malathion in environmental samples.

## 1. Introduction

Organophosphorus pesticides (OPs) are extensively used in modern agriculture to protect plants from insect and pest infestations. However, the widespread and long-term usage of pesticides has resulted in high quantities of residues of OP compounds in agricultural products, air, soil, and groundwater [1,2,3]. Due to their toxicity and potential carcinogenicity, the presence of pesticide residues in the environment could cause serious health problems in both humans and other living organisms [4,5]. The high toxicity of pesticides is mainly based on the irreversible inhibition of the enzyme acetylcholinesterase (AChE) [6,7,8]. This results in the accumulation of acetylcholine (ACh) neurotransmitters in the body, which causes cardiovascular and respiratory failure, as well as death [9,10]. Hence, the development of selective and sensitive methods for the detection of trace levels of pesticides in environmental samples is important.

Various conventional analytical methods, including chromatography [11,12,13,14,15], surface-enhanced Raman scattering [16], electrochemistry [17], colorimetry [18], and ultraviolet-vis spectroscopy [19], have been developed for monitoring pesticide residues. Although these methods have high sensitivity, as well as good selectivity and accuracy, they are cumbersome and require time-consuming sample pretreatment, expensive laboratory instruments, and highly qualified technicians. Thus, they are not suitable for on-site analysis [16,17,18].

In recent years, simple and novel liquid crystal (LC)-based biosensors have been developed for the determination of pesticide residues in environmental samples using AChE as a recognition element that is inhibited by pesticides [20,21,22]. The extraordinary physical properties of LCs, such as optical anisotropy, long-range orientational order, and responsiveness to external stimuli, make them a unique optical probe for monitoring biochemical events [23,24,25]. The working principle of these methods is mainly based on AChE inhibition by pesticides. Compared with the aforementioned methods, LC-based sensing methods offer many advantages such as low cost, rapidity, simplicity, and high sensitivity [26]. Moreover, LC-based biosensors have been considered promising portable sensing devices for practical application in the commercial market [27,28]. However, the lack of specificity of these methods remains a major challenge because almost all pesticides have inhibitory effects on AChE [16,18]. To enhance the specificity, aptamer-based biosensors have received considerable attention due to their excellent recognition ability to bind to various targets [29,30,31,32,33,34,35,36]. They offer many advantages, such as low cost, simple synthesis and modification, and high stability [37].

**Figure 1:**
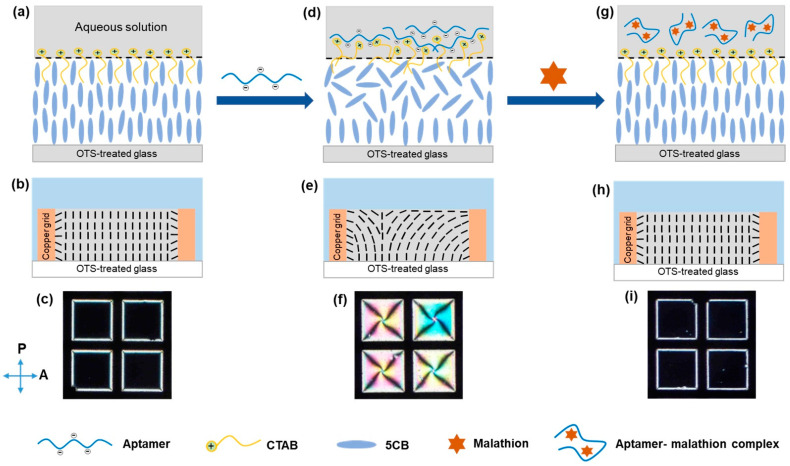
Schematic illustration of the orientational transition of LCs. (**a**) the self-assembly of CTAB at the aqueous/LC induces homeotropic orientation of LCs, (**d**) the interactions between CTAB and the aptamer at the interface disturb the homeotropic orientation of LCs, (**g**) upon binding with malathion, the conformational change of the aptamer renders the CTAB free to induce homeotropic alignment of LCs. (**b**,**e**,**h**): the corresponding schematic illustrations of the director fields of the LCs. (**c**,**f**,**i**): the corresponding polarized optical images associated with the orientation of LCs. OTS = *n*-octyltrichlorosilane.

Recently, LC-based biosensors using surfactant-decorated aqueous/LC interface have been widely developed for monitoring various biochemical events, such as enzymatic reactions [38,39], biomolecular interactions [40,41], and heavy metal ions [42,43]. In this paper, we constructed an LC-based sensing platform to detect malathion, a pesticide toxic to humans, in aqueous solutions. In our system, a DNA aptamer was employed as a molecular recognition probe for specifically binding to malathion, while CTAB, a cationic surfactant, was used to align LCs at the LC-aqueous interface in homeotropic orientation (Figure 1a), corresponding to a dark appearance of the LCs observed through a polarizing optical microscope (POM) (Figure 1c). In Figure 1c, the dark regions result from a homeotropic director of LCs throughout the LC thin film, while the bright white edges of the grid squares result from interactions between the LCs and the grid surface (as schematically illustrated in Figure 1b). In the absence of malathion, the addition of a negatively charged DNA aptamer disrupted the organization of the CTAB monolayer due to interactions between the aptamer and CTAB, leading to a planar alignment of the LCs at the interface (Figure 1d), resulting in a bright appearance of the LCs (Figure 1f). The bright appearance of the LCs in Figure 1f is a result of a continuous variation in the director field of LCs from a homeotropic to a planar orientation (as schematically illustrated in Figure 1e). However, in the presence of malathion, the formation of aptamer-malathion complexes caused a conformational change of the aptamers, thereby weakening the interactions between the aptamers and CTAB [20,44]. Therefore, CTAB remained available to align LCs at the interface in homeotropic orientation (Figure 1g). The dark appearance of the LCs in Figure 1i results from a homeotropic director of LCs throughout the LC thin film (as schematically illustrated in Figure 1h). The developed sensor exhibited a low detection limit (LOD) of 0.465 nM and excellent selectivity for malathion determination. In addition, the practical applicability of the developed sensing strategy has been demonstrated in real-world environmental samples, such as tap water, river water, and apples. Therefore, the proposed sensing method holds promises for simple, label-free, and highly sensitive detection of malathion and can be modified for the determination of other pesticides.

## 2. Materials and Methods

### 2.1. Materials and Apparatus

All the chemicals used were of analytical grade and the aqueous solutions were prepared with deionized (DI) water (resistivity of 18.2 MΩ.cm). Anhydrous n-heptane, CH_2_Cl_2_, H_2_SO_4_ (95 %), H_2_O_2_ (30%), CH_3_OH, and C_2_H_5_OH were procured from Daejung Chemicals & Metals Co., Ltd. of South Korea. Nematic liquid crystal 4-cyano-4’-pentylbiphenyl (5CB) was purchased from Tokyo Chemical Industrial Co., Ltd. of Japan. Malathion, ethion, fenthion, fenobucarb, carbofuran, phosmet, CTAB, phosphate-buffered saline (PBS, pH 7.4), and trichloro(octyl)silane (OTS) were obtained from Sigma-Aldrich, USA. The DNA aptamer with the sequence 5’-ATCC GTCA CACC TGCT CTTA TACA CAAT TGTT TTTC TCTT AACT TCTT GACT GCTG GTGT TGGC TCCC GTAT-3’ was synthesized by Mbiotech (Hanam, Korea). 

Polarized optical images of 5CB were captured using a digital camera (DS-2Mv, Nikon, Tokyo, Japan) affixed to a polarizing optical microscope (Eclipse LV100 POL, Nikon, Japan). All images were taken in transmission mode using a 4× objective lens. The gray-scale intensities (GIs) of the optical images were calculated using Adobe Photoshop CC2019 software (Adobe Inc., San Jose, CA, USA). Tests were conducted in at least triplicate for each measurement.

### 2.2. Preparation of OTS- Coated Glass Slides and Optical Cells

OTS-coated glass slides and optical cells were prepared as previously described [38,43]. The detailed procedures are provided in the Supplementary Material.

### 2.3. Detection of Malathion 

Malathion solutions of different concentrations were prepared in PBS. Aqueous aptamer solutions were incubated with malathion solutions for 2 h at 37 °C to obtain mixtures containing 200 nM aptamers and various concentrations of malathion. The mixed solutions of interest were then transferred into the optical cells containing a 4 µM CTAB-decorated LC interface and incubated for 30 min, followed by observation of the optical images under POM. Several commonly used pesticides, such as ethion, fenthion, fenobucarb, carbofuran, and phosmet, were used to test the specificity of the biosensor using similar procedures. To evaluate the practical applicability of the biosensor in real samples, the analysis was also carried out in tap water, river water, and apples. Tap water was collected from our laboratory, while river water was obtained from the Tancheon River, Seongnam City, Republic of Korea. The tap water and river water samples were filtered using a 0.22 µm-membrane filter to remove any suspended impurities. An apple was chosen as the matrix to test the applicability of the developed biosensor in food and was obtained from the local market (Seongnam city, Republic of Korea). The apple was cleaned with DI water, dried, and chopped into small pieces, followed by extraction with methanol for 2 h at room temperature (~25 °C). The extracts were filtered through a 0.22 µm-membrane filter to remove the solid part, and the solvent was evaporated, followed by dilution with PBS. The tap water, river water, and apple were spiked with different concentrations of malathion. The spiked real samples were then analyzed by applying the proposed assay.

## 3. Results and Discussion

### 3.1. Optimization of the Detection Conditions

Previous studies have demonstrated that CTAB could be used to regulate the orientation of the LCs at the LC- aqueous interface [42,43]. Therefore, the minimum concentration of CTAB sufficient enough to trigger the homeotropic anchoring of the LCs was first determined. For this, various concentrations of CTAB were added to LC thin film (thickness of ~20 µm) and incubated for 30 min. The polarized optical images of LCs at different concentrations of CTAB were then collected. As shown in Figure S1 (in the Supplementary Material), completely dark appearances of LCs were observed when the CTAB concentration ≥ 4 µM (Figure S1e,f), corresponding to the homeotropic anchoring of the LCs at the interface. Therefore, a CTAB concentration of 4 µM was employed in subsequent experiments.

**Figure 2:**
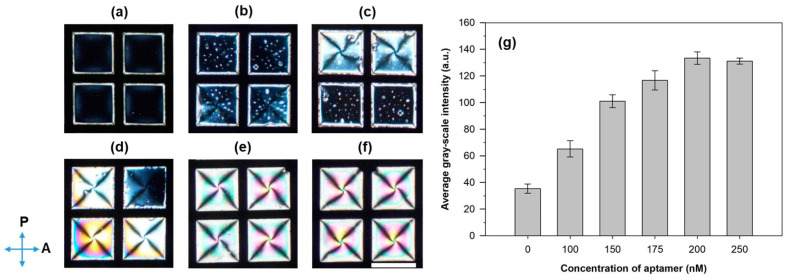
Polarized optical images of the CTAB-decorated aqueous/LC interfaces after 30 min in contact with various aptamer concentrations: (**a**) 0 (only PBS), (**b**) 100, (**c**) 150, (**d**) 175, (**e**) 200, and (**f**) 250 nM. (**g**) The corresponding average GIs of the polarized optical images. The direction of the polarizer and analyzer is indicated by blue arrows. Scale bar, 285 µm.

Next, the optical behavior of the CTAB-decorated aqueous/LC interfaces after adding aqueous solutions of the malathion aptamer was investigated. After incubation with CTAB solution for 30 min, excess CTAB in the optical cell was removed and then 200 µL of aptamer solutions at different concentrations were added. LC thin film displayed a dark optical appearance when in contact with PBS solution (Figure 2a), suggesting that the PBS solution did not alter the orientational alignment of the LCs at the interface. At 100 nM concentration of aptamer (Figure 2b), the nucleation of small birefringent domains was observed. At aptamer concentrations ranging from 150 to 175 nM (Figure 2c,d), the small birefringent domains grew and coalesced to form larger birefringent domains, however, the optical appearances of LCs were not fully bright. For concentrations of aptamer ≥200 nM (Figure 2e,f), the optical appearances of LCs became completely bright, which corresponded to a planar/tilted alignment of LCs at the aqueous/LC interface. These results indicated that the LC molecules undergo an orientational transition from homeotropic to planar/tilted orientation with the increase in the aptamer concentration. This is due to the electrostatic interactions between the cationic head groups of CTAB and the anionic phosphate groups of the DNA aptamer disrupting the homeotropic alignment [41,42,43]. Moreover, the average GI values of the corresponding optical images gradually increased with increasing aptamer concentration and reached a maximum value at the aptamer concentration of 200 nM (Figure 2g). Thus, 200 nM aptamer was used for the detection of malathion in subsequent experiments.

### 3.2. Specificity of the Developed LC Biosensor

**Figure 3:**
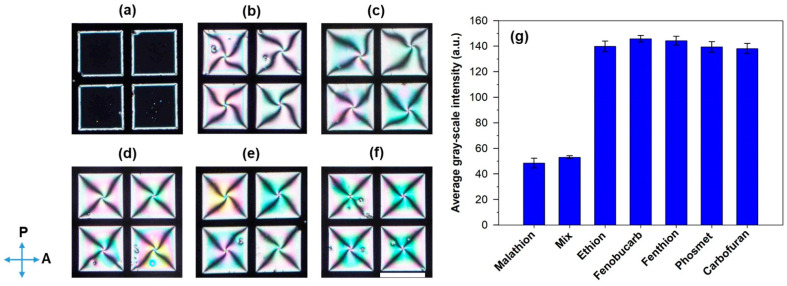
Polarized optical images of the CTAB-decorated aqueous/LC interfaces after 30 min in contact with pre-incubated mixtures containing 200 nM malathion aptamers and different pesticides: (**a**) malathion, (**b**) ethion, (**c**) fenobucarb, (**d**) fenthion, (**e**) phosmet, and (**f**) carbofuran. (**g**) The corresponding average GIs of the polarized optical images. The direction of the polarizer and analyzer is indicated by blue arrows. Scale bar, 285 µm.

The specificity of the developed biosensor for malathion detection was studied by investigating the orientational transition of LCs in contact with the pre-incubated mixtures containing 200 nM aptamers with malathion or other interfering pesticides, such as ethion, fenthion, fenobucarb, carbofuran, and phosmet [16,18]. In the experiment, the concentration of malathion was maintained at 600 nM, while the interfering pesticides were maintained at 1 μM. As shown in Figure 3a, a completely dark appearance of LCs was observed when 200 nM aptamers were incubated with malathion. In contrast, bright appearances of LCs were observed when 200 nM aptamers were incubated with other pesticides (Figure 3b–f). In addition, the average GI values of the optical images for malathion and the mixture of malathion and interfering pesticides were significantly lower than that of other interfering pesticides (Figure 3g and Table 1). Moreover, it was found that the presence of the interfering pesticides did not significantly affect the GI value of the optical images for malathion. These results suggest excellent specificity of the developed LC biosensor for malathion.

### 3.3. Analytical Performance of the LC-Based Sensing Method

**Figure 4:**
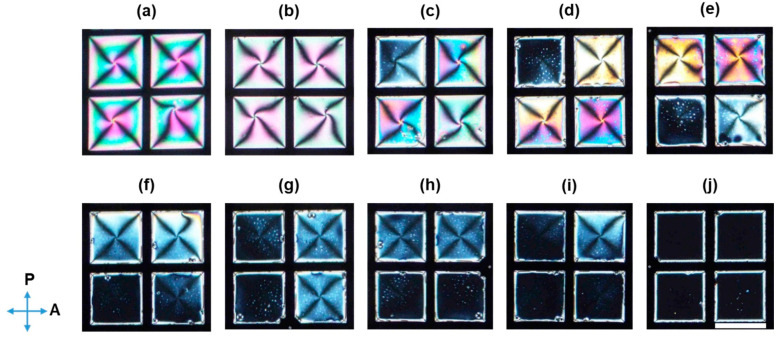
Polarized optical images of the CTAB-decorated aqueous/LC interfaces after 30 min in contact with pre-incubated mixtures containing 200 nM malathion aptamers and different concentrations of malathion: (**a**) 0, (**b**) 1, (**c**) 3, (**d**) 5, (**e**) 10, (**f**) 50, (**g**) 100, (**h**) 200, (**i**) 400, and (**j**) 600 nM. The direction of the polarizer and analyzer is indicated by blue arrows. Scale bar, 285 µm.

The qualitative performance of the developed LC biosensor in the detection of malathion was tested by investigating the orientational transition of LCs in contact with the pre-incubated mixtures containing 200 nM aptamers with various concentrations of malathion. The LC patterns gradually changed from bright to dark with increasing concentration of malathion from 1 to 600 nM (Figure 4), corresponding to an ordering transition from planar to homeotropic of LCs. The higher the concentration of malathion in the sample, the darker the obtained optical image.

**Figure 5:**
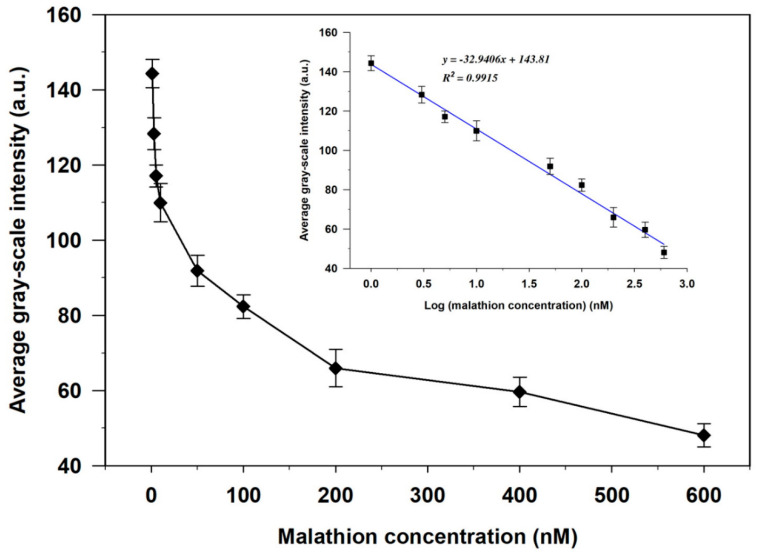
Relationship between the average GIs of the optical images and malathion concentrations. Inset shows a linear relationship between the average GI and the logarithmic concentration of malathion.

The quantitative performance of the developed biosensor was further investigated by evaluating the relationship between the average GIs of the polarized optical images and the malathion concentration. As shown in Figure 5 and Table 2, the average GIs of the optical images gradually decreased with increasing malathion concentration from 1 to 600 nM. Low relative standard deviations (RSD) ranging from 2.94 to 5.1% were obtained (Table 2), indicating the high repeatability of the developed sensing system. The highest sensitivity of 8.001 nM^−1^ was attained (Table S1 in the Supplementary material). A good linear relationship between the average GIs and the logarithmic concentration of malathion was observed in this concentration range, with a linear correlation coefficient (R^2^) of 0.9915 (Figure 5, inset). The detection limit of malathion was calculated using the formula 3α/slope [18,45] and was found to be 0.465 nM. The results indicate that this biosensor can potentially be applied for qualitative and quantitative detection of malathion in aqueous solutions.

### 3.4. Real Sample Analysis with the Proposed LC Biosensor

To evaluate the applicability of the proposed biosensor in real samples, the analysis was carried out in tap water, river water, and apples. Different concentrations of standard malathion were spiked into real samples. The spiked real samples were then analyzed by applying the proposed assay to investigate the recoveries. The recovery values of real samples ranging from 87.96% to 110.23% with a relative standard deviation of less than 9% were obtained (Table S2 in the Supplementary material), indicating the high accuracy of the developed biosensor for malathion detection and the low matrix effect on the optical response of the LCs. Therefore, this LC-based aptamer sensing platform can be successfully applied for the determination of malathion in actual samples.

Before concluding, we compare the analytical performances of the proposed LC-based sensing method with different detection techniques such as electrochemical, colorimetric, amperometric, and chromatographic for the detection of malathion. The proposed method exhibited a wide linear range and a low detection limit of 0.465 nM, which is comparable to or lower than those of the recently reported detection techniques (Table 3). In addition, this method does not require the labeling of the analytes, the use of organic solvents, and costly equipment. In contrast, the reported detection methods require sophisticated and expensive laboratory instruments, time-consuming sample pretreatment, and highly qualified technicians, thus, they are not suitable for on-site analysis. Moreover, the use of DNA aptamers as molecular recognition probes has improved the selectivity of the proposed method towards malathion. Therefore, compared with other detection techniques (such as electrochemical, colorimetric, and chromatographic), the proposed LC-based sensing method offers many advantages such as simplicity, rapidity, low cost, high sensitivity, high selectivity, label-free, and on-site detection.

## 4. Conclusions

In summary, we developed a simple and label-free LC biosensor based on a cationic surfactant-decorated LC interface for the detection of malathion, a pesticide toxic to humans. LCs displayed a bright appearance when an aqueous aptamer solution was introduced onto the CTAB-decorated LC interface. However, a dark appearance of LCs was observed when a pre-incubated mixture of the aptamer and malathion was transferred onto the CTAB-decorated LC interface. The combination of a highly specific aptamer technology and a highly sensitive liquid crystal-based sensing method provides a novel sensing strategy for the effective detection of malathion which improves sensitivity and selectivity. The proposed biosensor has high specificity for malathion and high sensitivity with a detection limit as low as 0.465 nM. Moreover, the developed biosensor was successfully applied for the detection of malathion in environmental samples, including tap water, river water, and apples, with satisfactory results. Therefore, the proposed sensing method holds promises for simple, label-free, highly sensitive, and highly selective detection of malathion and can be modified for the detection of other pesticides in environmental samples.

## Figures and Tables

**Table 1:** The average gray-scale intensity (AGI) values of the optical images obtained after the CTAB-decorated aqueous/LC interfaces were in contact with pre-incubated mixtures containing 200 nM malathion aptamers and different pesticides for 30 min.

Pesticides	AGI (a.u.)	RSD ^a^
Malathion	48.54	3.75
Ethion	139.82	4.15
Fenobucarb	145.81	2.54
Fenthion	144.26	3.46
Phosmet	139.39	4.1
Carbofuran	138.14	3.95
Mix ^b^	53.05	1.11

^a^ RSD, relative standard deviation. ^b^ Mix, mixture of malathion and other interfering pesticides.

**Table 2:** The AGI values of the optical images obtained after the CTAB-decorated aqueous/LC interfaces were in contact with pre-incubated mixtures containing 200 nM aptamer and different concentrations of malathion for 30 min.

Malathion concentration (nM)	AGI (a.u.)	RSD
1	144.314	3.75
3	128.312	4.21
5	117.08	2.94
10	109.946	5.1
50	91.884	4.16
100	82.344	3.15
200	65.97	4.97
400	59.62	3.87
600	48.106	3.12

**Table 3:** Comparison of the proposed assay with the available methods for malathion detection.

Method (Material)	Detection Range	Detection Limit	Reference
Colorimetric (AuNPs) ^a^	50–800 nM	11.8 nM	[4]
GC-FPD ^b^	30.27 pM–302.7 nM	24.2 pM	[11]
GC-TSD ^c^	0.01–12.0 mg/kg	0.3 mg/kg	[12]
SERS ^d^	9.99–100.8 μM	10 μM	[46]
Colorimetric (Pd@AuNRs) ^e^	0.001–200 μg/mL	181 nM	[47]
Amperometry (PAn-PPy-MWCNTs) ^f^	0.03–75.67 μM	3.03 nM	[48]
Electrochemical (PTT) ^g^	9.99–99.01 nM	4.08 nM	[49]
UV-Vis spectroscopy (AgNPs) ^h^	0–100 nM	0.455 nM	[50]
Electrochemical (PA6/PPy/RGO) ^i^	1.51–60.54 μM	2.42 nM	[51]
Liquid crystal	1–600 nM	0.465 nM	Present study

^a^ AuNPs, gold nanoparticles (NPs). ^b^ GC-FPD, gas chromatography-flame photometric detection. ^c^ GC-TSD, gas chromatography-thermionic specific detection. ^d^ SERS, surface-enhanced Raman spectroscopy. ^e^ Pd@AuNRs, palladium-gold nanorods. ^f^ PAn-PPy-MWCNTs, polyaniline/polypyrrole copolymer doped with multi-walled carbon nanotubes. ^g^ PTT, poly [2,2′;5′,2″]-terthiophene-3′-carbaldehyde. ^h^ AgNPs, silver NPs. ^i^ PA6/PPy/RGO, polyamide 6/polypyrrole electrospun nanofibers coated with reduced graphene oxide.

## Data Availability

Not applicable.

## Supplementary Materials

The following are available online at https://www.mdpi.com/2079-6374/11/3/92/s1, Figure S1: Polarized optical images of the aqueous/LC interfaces after 30 min in contact with CTAB solutions at different concentrations: (**a**) 0 (only PBS), (**b**) 1, (**c**) 2, (**d**) 3, (**e**) 4, and (**f**) 5 µM. The direction of the polarizer and analyzer is indicated by blue arrows. Scale bar, 285 µm; Table S1: Detection of malathion in spiked real samples applying the proposed assay (n = 3).
