# Supplementary material for: A Cationic Surfactant-Decorated Liquid Crystal-Based Aptasensor for Label-Free Detection of Malathion Pesticides in Environmental Samples
Source: Biosensors (Basel). 2021 Mar 23;11(3):92. doi: 10.3390/bios11030092 (PMC8004806; doi:10.3390/bios11030092)
Supplement: Supplementary file 1 [file biosensors-11-00092-s001.pdf]

Supplementary Material

A cationic surfactant-decorated liquid crystal-based aptasensor for label-free detection of malathion pesticides in environmental samples

Duy Khiem Nguyen[1], and Chang-Hyun Jang[1,*]

[1]Department of Chemistry, Gachon University, Seongnam-daero 1342, Sujeong-gu, Seongnam-si, Gyeonggi-do 13120, Republic of Korea.

[*]Corresponding author: Tel. +82-31-750-8555; fax: +82 31 750 8774

E-mail address: chjang4u@gachon.ac.kr (C.-H. Jang)

Preparation of OTS- coated glass slides and optical cells

First, the glass slides were cleaned with "piranha solution" (70% H_2SO_4 and 30% H_2O_2, v/v) for 1 h at 80 °C. The glass slides were then rinsed with DI water, ethanol, and methanol, and then dried under a stream of gaseous N_2, followed by heating at 120 °C for at least 3 h. After cooling, the cleaned glass slides were immersed in an OTS/heptane solution for 40 min at room temperature (~25 °C). After washing with methylene chloride, the OTS-treated glass slides were dried under a stream of N_2 and stored in a desiccator until further use.

The OTS-treated glass slides were fixed to the bottom of an eight-well chamber slide using silicone glue. On each well, the cleaned copper TEM grids were placed onto the OTS-treated glass slides. The TEM grids were then filled with approximately 1 μL of 5CB, and the excess 5CB was removed with the help of a 20 μL capillary tube to produce a uniform LC thin film with a thickness of approximately 20 μm. Finally, 200 μL of aqueous solutions of interest were introduced into the optical cell using a micropipette at room temperature (~25 °C). All experiments were repeated at least three times. After use, the optical cells and copper TEM grids were washed with DI water and ethanol, and then dried in an oven at 40 °C. The optical cells and copper TEM grids can be reused at least five times without significant effect.

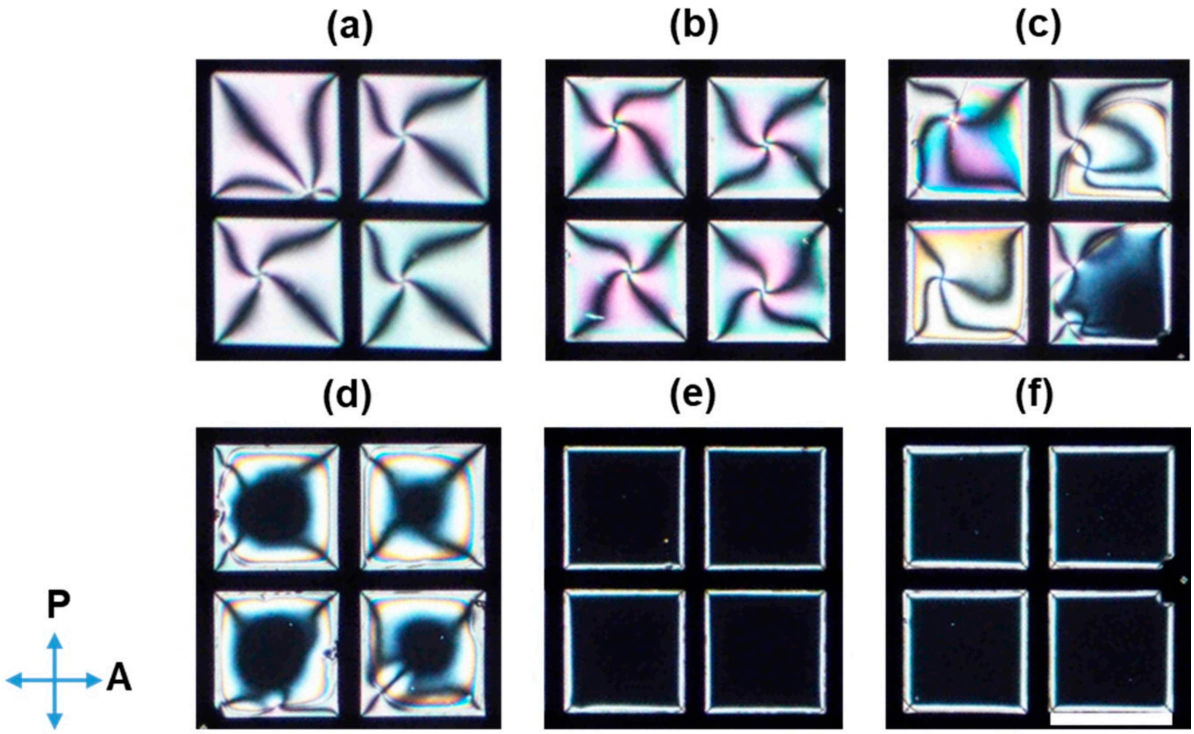

Figure S1. Polarized optical images of the aqueous/LC interfaces after 30 min in contact with CTAB solutions at different concentrations: (a) 0 (only PBS), (b) 1, (c) 2, (d) 3, (e) 4, and (f) 5 μM. The direction of the polarizer and analyzer is indicated by blue arrows. Scale bar, 285 μm.

Table S1 Detection of malathion in spiked real samples applying the proposed assay (n = 3).

Samples	Added (nM)	Found (nM)	Recovery (%)	RSD (%)
Tap water 1	10	8.872	88.72	5.08
Tap water 2	50	48.35	96.7	4.71
Tap water 3	75	68.69	91.59	8.32
Tap water 4	100	105.26	105.26	2.37
Tap water 5	150	165.34	110.23	7.26
Tap water 6	200	195.64	97.82	6.97
Tap water 7	300	267.21	89.07	2.3
Tap water 8	400	409.56	102.39	8.41
Tap water 9	500	490.59	98.12	3.15
Tap water 10	600	527.76	87.96	5.65
River water 1	10	9.125	91.25	7.09
River water 2	50	49.123	98.25	4.76
River water 3	75	69.12	92.16	3.43
River water 4	100	95.61	95.61	5.91
River water 5	150	161.42	107.61	6.25
River water 6	200	211.62	105.81	4.96
River water 7	300	272.7	90.9	1.68
River water 8	400	391.88	97.97	2.1
River water 9	500	465.816	93.16	5.18
River water 10	600	537.6	89.6	7.09
Apple 1	10	10.157	101.57	8.74
Apple 2	50	49.18	98.36	4.14
Apple 3	75	79.38	105.84	6.66
Apple 4	100	94.76	94.76	3.59
Apple 5	150	136.515	91.01	4.03
Apple 6	200	201.12	100.56	6.98
Apple 7	300	294.15	98.05	8.03
Apple 8	400	435.16	108.79	4.39
Apple 9	500	516.438	103.29	5.23
Apple 10	600	546.6	91.1	5.18
